# Supplementary material for: Have Policies Tackled Gender Inequalities in Health? A Scoping Review
Source: Int J Environ Res Public Health. 2021 Jan 5;18(1):327. doi: 10.3390/ijerph18010327 (PMC7796005; doi:10.3390/ijerph18010327)
Supplement: Supplementary file 1 [file ijerph-18-00327-s001.zip › Supplementary files 1,2 and 3/Supplementary File 1. Search Strategies.docx]

**Supplementary File 1. Search Strategies.**

**Date of searches: 15th May 2019**.

| **Database #1. Medline through Pubmed: 950 items found** |
| --- |
| (health system*[Title/Abstract] OR community health[Title/Abstract] OR health policy[Title/Abstract] OR health policies[Title/Abstract] OR health service*[Title/Abstract] OR sanitary system*[Title/Abstract] OR politic*[Title/Abstract])) AND gender[Title])*Filters:*Clinical Trial; Journal Article; Review; Technical Report; Classical Article; Humans; English; French; Portuguese; Spanish |
| **Database # 2: Web of science (WOS) and SCIELO: 2551 items found** |
| TS=("health system*" OR "community health" OR "health policy" OR "health policies" OR "health service*" OR "sanitary system*") AND TS=(health) AND TI=(gender*)  *Filters:*  Article or clinical trial or review or report; English or Spanish or Portuguese or French |
