# Supplementary material for: Have Policies Tackled Gender Inequalities in Health? A Scoping Review
Source: Int J Environ Res Public Health. 2021 Jan 5;18(1):327. doi: 10.3390/ijerph18010327 (PMC7796005; doi:10.3390/ijerph18010327)
Supplement: Supplementary file 1 [file ijerph-18-00327-s001.zip › Supplementary files 1,2 and 3/Supplementary File 2. Policies.docx]

| **1st author, year** | **Aim** | **Methods** | **Time frame; location** | **Policy area** | **Health problem** | **Analysis** | **Policies** |
| --- | --- | --- | --- | --- | --- | --- | --- |
| **Amroussia, 2016** [18] | (1)To explore the extent to which reproductive rights have been incorporated into the country’s reproductive health policy in Tunisia; (2) to determine gaps in the implementation of this policy; and (3) to examine how the gender empowerment process has been influenced by this policy | Qualitative. Document analysis applying a HeRWAI adapted tool. Different sources: Literature reviews, official data, reports | 1994-2014;  Tunisia | Reproductive health policies and (Health policies) | Reproductive health | Policy formulation;  Policy implementation; Policy evaluation | National reproductive health policy (plans, programs and strategies such as national health programs, family planning program, antenatal program, national program to combat STD and HIV). Also laws related to reproductive rights. Examples of the rights: legal abortion, use of contraceptive methods, equity during marriage and divorce proceedings, the right to interracial, interreligious and civil marriage, the right to reproductive health |
| **Arístegui, 2017** [19] | To describe the initial and qualitative results of a larger study that aimed to: (1) to investigate the impact of the implementation of the Argentinean Gender Identity Law on the life conditions of the transgender population and (2) to identify perceived improvements and barriers to the law’s implementation | Qualitative. Focus groups | 2013;  Argentina | Gender identity policies (No health policies) | Health of transgender people | Policy implementation; Policy evaluation | Argentinean Gender Identity Law promotes  Human rights and equality between transgender individuals and the rest of the Argentinean population by recognizing the right to a self-defined gender identity.  It allows individuals to change their names and gender on birth certificates, previously granted educational diplomas and awards, and on all government issued forms of identification. Gender recognition is guaranteed without arbitrary reconditions and barriers. This is a very straightforward administrative process that does not require any kind of proof of partial or total genital reassignment surgery, hormonal therapies, or any other psychological or medical treatment nor the need to have psychiatric or judicial approval |
| **Backhans, 2012** [20] | To investigate the link between gender policy and the gender gap in cause-specific mortality, adjusted for economic factors and health behaviours | Quantitative. Ecological study. Association analysis | 1973-2008;  Global. 22 OECD countries | Family or labour models (No health policies) | External cause and circulatory disease mortality | Policy evaluation | Gender policy regimes, that were established regarding: parental leaves, social services, separate taxation, pension universality, monetary support to breadwinner and compensatory measures in pension system |
| **Baizan,**  **2016** [21] | To assess the extent to which individual-level completed fertility varies across contexts characterized by policies supporting different gender division of labour models | Quantitative. Association analysis. Individual-level data from the European Union Survey on Income and Living Conditions, combined with country-level data | 2004-2009;  Europe: 16 Western and Southern European countries | Family or labour models (No health policies) | Fertility | Policy evaluation | Labour market and care policies, specifically: family allowances,  weighted leave weeks,  childcare coverage,  childcare usage, men working hours, % women on part-time |
| **Billingsley, 2014** [22] | To analyse variation in fertility intentions of 16,000 men and women according to individual-level characteristics and family policy across 21 European countries. | Quantitative. Ecological study. Policy data: multiple sources; Fertility intention data: European Social Survey in 2004 | Until 2000 the policies, from 2004 the fertility intentions; Europe. 21 countries | Family or labour models (No health policies) | Fertility | Policy implementation | Policies related to the two family or labour models used in the study:  a) Traditional model: child allowances, tax subsidies, marriage subsidies, home-care allowance  b) Earner- carer model: earnings-related leave, childcare provision |
| **Borrell,**  **2014** [23] | 1) To examine the influence of macrosocial policies on gender inequalities in health or on women’s health and 2) to provide an example of published evidence on the effects of a specific policy (parental leave) on women’s health | Quantitative. Ecological study. Association analysis by using between-country (or administrative units within a country) comparisons | 1970-2012;  Global but mainly USA and Europe | Macrosocial policies, including family or labour models (No health policies) | Women's general health and health inequalities | Policy evaluation | Many different macrosocial policies, including: family and labour policies, policies to promote political representation, gender equitable use of time and equal incomes |
| **Burke,**  **2017** [24] | To exemplify how a community coalition can develop and incorporate strategies for addressing gender and social connectedness through community health improvement initiatives and demonstrate how it can lead to policy, systems, and environmental changes to improve gender norms and positively impact women’s health. | Mixed methods.  Case study | 2010-2015;  City of Inkster, Michigan, USA | Any kind of policy (No health policies) | Health | Policy implementation | Establish collaborations. In addition, changes at the organizational level that range from documented agreements to improvements that reinforce healthy choices. The modifications were intended to achieve sustainability over time. Specific examples: creating the job position of community health worker; Establish agreements with a local church to use its facilities, provide a mobile health van to provide health screenings to residents; arrange a hospital collaboration on the provision of antenatal services. |
| **Carael,**  **2009** [25] | To summarize data on policies and strategies affecting women and men equity in access to antiretroviral treatment and other HIV services, as reported by countries | Quantitative. Sources: Mainly data from National Composite Policy Index, other core UNGASS indicators and national household surveys. | 2003-2008;  Global. 130 United Nations Member States | Health policies and any other kind of policies (Health and No health policies) | HIV and IPV-related health | Policy formulation; Policy implementation; Policy evaluation | Any kind of policies, including health policies, strategic plans, laws and regulations, affecting women and men equity in access to antiretroviral treatment and other HIV services. Also policies addressing violence against women and achieving greater involvement of men in reproductive health programs. And also policies for boosting women’s economic empowerment or countering gender norms that increase women’s vulnerability to HIV |
| **Ewig,**  **2012** [26] | To evaluate the effectiveness of the specific reforms directed at Chilean private health providers (2004 ) that sought to reduce gender and age inequality | Quantitative. Gender and age equity comparisons in access to private health care before and after the health reforms. Demographic comparisons and regression analysis. Sources: Databases | 1998-2009;  Chile | Health care policies (Health policies) | Health | Policy evaluation | Policies were laws to reform the health sector, intended to decrease inequalities in access to health care. Two of the new laws specifically addressed discrimination by private insurers and its resultant inequality, and increasing equity and quality of health services in the public sector |
| **Gavriilidis, 2014** [28] | To pilot-evaluate the GEIDP’s (gender equity integration development plan) potential for empowerment and to test the PEI (Policy Empowerment Index) for future policy evaluations | Mixed methods. Design of a tool and its subsequent application to analyse an equity plan. Sources: analysis of documents and statistics | Unspecified. Approx. 2012- 2013;  City of Malmo, Sweden | Gender policies (No health policies) | Health | Policy formulation; Policy implementation | A Plan for gender equity integration including a wide diversity of policies: policies related to increasing grassroots and interest groups participation in planning, agenda setting, design; policies related to training on gender equity among city employees and students; policies for creating and strengthening jobs and business opportunities, policies for empowering vulnerable populations |
| **Gideon,**  **2006** [29] | To review initiatives to integrate gender interest into health policy in Chile. Mainly focused on the “Mainstreaming Gender Equity in Health Sector Reform Programme” promoted by the Panamerican Health Association. | Qualitative. Semi-structured interviews with activists and professionals involved in the programme | 2000-2003;  Chile | Gender mainstreaming in health sector reform (Health policies) | Health | Policy formulation; Policy implementation | The “Mainstreaming Gender Equity in the Health Sector Reform Programme”, was developed as a response to national and international demands by the PAHO. Initially the programme was conceived by the Women in Development Unit within the PAHO in Washington, in consultation with health activists in Latin America and Chile was selected as one of the pilot countries. Objectives:  a) to increase to increase women’s voice in policy-making and to produce statistical information about levels and tendencies of gender inequalities in health that women can use to inform their campaigns  b) to improve accountability and transparency of gender policies |
| **Gill,**  **2011** [30] | To compare current South Asian women’s health status given the existence or nonexistence of gender equitable policies and assess the implications for implementation and monitoring of existing policies | Quantitative. Comparison of gender-sensitive indices by country according to the existence or non-existence of a certain policy | 1996-2008;  South Asia: Bangladesh, India, Nepal, Pakistan, and Sri Lanka | Laws, educational policies, policies related to political and economic empowerment (No health policies) | Health | Policy evaluation | Different types of policies including regulations protecting women’s rights and women’s health. For example, regulations and laws protecting women form violence (IPV, trafficking, child marriages, sex selection), regulations for protecting sexual and reproductive rights of women.  Also educational policies and policies that promote political and economic empowerment of women, etc. |
| **Hardee,**  **2014** [32] | (1)To examine the evidence related to six key social and structural drivers of HIV for women (gender norms, violence, legal norms, employment, education, stigma and discrimination). (2) To review the evidence for successful and promising social and structural interventions related to each driver | Systematic review. Sub-analysis based on a broader systematic review | 2005-2011;  Global | Policies aimed at addressing social and structural drivers and outcomes on gender inequalities in HIV. (No health policies) | HIV and IPV-related health | Policy evaluation | Wide range of actions to strengthen the enabling environment for women and girls focused on transforming gender norms, addressing violence against women, transforming legal norms, promoting women’s employment, income and livelihood opportunities, advancing education and reducing stigma and discrimination |
| **Hendriks, 2018** [33] | To assess current Belgian health services in the context of international guidelines and standards and to evaluate the degree to which the implementation of the Belgian Sexual Assault Care Centre model could contribute to both a more integrated and gender-sensitive care delivery for victims of sexual assault | Mixed methods. Survey of professionals and comparison with international guidelines and standards SWOT (Strengths, Weaknesses, Opportunities, Threats) analysis | 2017-2018;  Belgium | Health care policies (Health policies) | Sexual assault/ IPV | Policy implementation; Policy evaluation | Sexual assault health services in potential Belgian Sexual Assault Care Centre hospitals: sexual assault care protocols; gender training for health professionals; services offered (follow up, integration of care, accessibility of care), infrastructure and equipment |
| **Hills,**  **2002** [34] | Unclear objective but could be summarized as: to analyse the documents and guiding principles of associations and modify them to make them more gender-sensitive. (2) to develop strategies for the incorporation and endorsement of them and facilitate its implementation | Qualitative. Co-operative inquiry that brought together 10 regional health professional associations | Unspecified. Approx. 2000;  British Columbia province, Canada | Health policies | Health | Policy formulation | Policies: Inclusion of gender sensitivity in the guiding principles and documents of the associations |
| **Jonsson, 2006** [35] | To present the major findings of the Board’s* (National board of Health and Welfare) follow-up inquiry, whose objective was to review and analyse gender equity trends in health care, and to discuss conclusions to be drawn on how to promote a more gender sensitive and equitable health care | Mixed methods. Compares the situation in health after the 1996 proposals on gender and health. Data from multiple sources: surveys, meetings, reports, health and quality registers, literature review, etc. | 1996-2004;  Sweden | Health care, health education and health research policies. (Health policies) | Health | Policy evaluation | Different health care policies as well as health education and health research policies. For example: training for health professionals, requirements and strategies for inclusion of gender perspective in research and for monitoring gender sensitivity in healthcare system. Also policies related to reporting gender statistics or the inclusion of a gender perspective into the formulation of health policy documents in county councils |
| **Keippel, 2017** [36] | To examine and describe how a community coalition, Healthy By Design, infused a gender focus into the policymaking process | Qualitative. Case study. Focus groups | 2011-2016;  Yellowstone County, Montana, USA | Street safety policies (No health policies) | Related to physical activity, social cohesion, etc. | Policy implementation | Policies related to street safety, which in turn influence women's health through facilitating physical activity and social connectivity |
| **Kuhlmann, 2012** [37] | To explore how gender mainstreaming is translated into the institutional configurations of the healthcare systems in England and Germany | Qualitative. Scoping review and case studies. Multiple sources: document analysis, other secondary sources and additional expert information | Approx. 2000-2009; England and Germany | Health care policies (Health policies) | Health | Policy implementation | Analysis of the gender mainstreaming in England and Germany. Strategies on the management in the field of primary care for chronic diseases, namely the National Service Frameworks in England and the Disease Management Programmes in Germany |
| **Lamprell, 2017** [38] | (1) To assess whether, the extent to which, and how gender has been represented in PNG’s² health sector policy, and the themes and concepts as they sit embedded in policy documents and frameworks. Then (2), armed with this analysis, a set of recommendations for strengthening gender equity for the benefit of PNG’s health system are made | Quantitative. Document analysis (policy core documents) using both data-mining and traditional, summative content analysis | 2015; Papua New Guinea (PNG) | Health policies | Health | Policy formulation;  Policy implementation | Inclusion and conceptualization of gender in health sector policy documents and frameworks |
| **Minvielle, 2011** [39] | (1) To explore the incorporation of women’s right to health into the main health policy documents of St. Lucia, St. Vincent and the Grenadines, and Guyana. (2)To make a comparative assessment of government commitment, focusing on intimate partner violence and access to legal abortion | Qualitative. Document analysis and comparative analysis was conducted based on the application of the HeRWAI to the health plans. Sources: health plans, budget addresses, Constitutions, reports, mainly from WHO and CEDAW⁵ | Until 2010; Countries in the Anglo-Caribbean: St. Lucia, St. Vincent and the Grenadines and Guyana | Mainly the health plan, legislative and awareness policies. (Health and No healh poliies) | SRH and IPV-related health | Policy formulation; Policy implementation | The formulation of health plans, budget allocation for gender policies. Also laws and regulations to protect women’s sexual and reproductive rights (promotion of healthy sexuality, access to safe abortion, family planning) and awareness policies related to IPV |
| **Olinyk,**  **2014** [40] | To explore the perspectives of those involved in developing and implementing the "Agenda for accelerated country level action on women, girls, gender equality and HIV", to understand its strengths and limitations | Qualitative. Case studies. In-depth one-on-one interviews with professionals involved in the development and implementation of the Agenda | 2010-2011; Global | Gender and HIV policies (Health and No health policies) | HIV-related health | Policy implementation | The "Agenda for accelerated country level action on women, girls, gender equality and HIV", an operational plan to provide guidance, frameworks and mechanisms for integrating gender in regional and national HIV policies |
| **Panisello, 2016** [41] | To identify and analyse progress and difficulties in the development of the gender perspective in health policies in Catalonia | Qualitative: document analysis (health plans, public healh plan, legislative measures and gender-sensitive government actions) and interviews with professionals | 2006-2015;  Catalonia, Spain | Health policies | Health | Policy formulation; Policy implementation | The study analysed policies in Catalonia, mainly The Catalonian Health Plan, Public Health Plan and several Directive Plans related to health.  The study also analysed the implementation of gender-sensitive health policies from the point of view of professionals |
| **Payne,**  **2015** [42] | To explore the limited progress of gender equality policies, through a study of the local implementation of equalities policies and explore explanations of the implementation gap between policy and practice. The authors also highlight the role of equalities leads in the public sector as local “agents of change” | Qualitative exploratory. Semi-structured interviews with equality leaders from 9 health organisations | 2012; UK | Health sector policies (NHS) (Health policies) | Health | Policy implementation | Gender-sensitive policies in 9 public-sector organisations in the NHS. The study analysed the implementation of gender equality policies. No specific policy details are given |
| **Rottach,**  **2017** [43] | To deconstruct the various dimensions of gender and identify how programmes address these domains to improve health | Systematic review. Sub-analysis based on a broader systematic review | Not specified Approx. until 2013-2014; Asia, Middle East, sub-Saharan Africa and Latin America | Policies/ programmes aimed mainly to addressing gender norms, power dynamics, legal status and rights and access to health resources (No health policies) | Family planning, or maternal or child health | Policy formulation;  Policy evaluation | Programmes that used gender approaches to achieve a range of gender and health goals. Gender-transformative programmes: typically pursued gender equality goals, such as changing rigid gender roles or increasing women’s autonomy. Accommodating programmes more commonly used gender strategies to achieve health-specific goals, like increasing contraceptive use or improving child nutrition. |
| **Sen,**  **2014** [44] | Not specified. It could be summarised as follows: To analyse the formulation of the United Nations Millennium Development Goals in relation to gender equity in relation to its human rights-based approach and its support for the women's movement to activate and energize the the post-2015 Development Agenda | Qualitative. Document analysis | Not specified. Approx 1996-2002;  Global. The 193 Member States of the United Nations | Gender policies (No health policies) | Health | Policy formulation | The United Nations Millennium Development Goals, specifically Millenium Development Goal 3 “Promote gender equality and women’s empowerment”: the set of goals, targets, indicators. Also the related politics of agenda setting and the support for women's organisations |
| **Stewart,**  **2009** [48] | To assess the extent of gender mainstreaming in health policy in a low- (Peru) middle (Colombia), and high (Canada)- income country in the Americas | Qualitative. Selection and analysis of indicators (policies) and comparisons between countries. Data from multiple sources (digital sources, documents and consultation with experts or ONG's) | 2000-2006;  Peru, Colombia and Canada | Mainly laws and other regulations (No Health policies) | Health | Policy formulation; | Mainly laws and regulatory measures related to sexual and reproductive rights, gender-based violence, family planning, promotion of gender equality in the media, and maternity and paternity leave, laws against gender discrimination. Also other indicators (policies) related to the same areas, including health plans, guidelines or accessibility of sexual and reproductive health services |
| **Theobald, 2005** [50] | To explore the impetus for and process of gender mainstreaming in SWAPs⁶ in the Ministries of Health in Uganda, Ghana, Malawi and Mozambique, and to outline some achievements and challenges. | Qualitative. Workshop for Ministerial gender focal points and NGO representatives working on mainstreaming gender in SWAPs | Not specified. Approx. 2003;  Africa: Uganda, Ghana, Malawi and Mozambique | Health sector policies (Health policies) | Health | Policy implementation | Gender-sensitive policies in the health sector in the context of SWAps. SWAps are an approach to aid where government takes the  lead in developing a coherent policy and expenditure programme for a particular sector. Donors work in partnership with government and civil society organizations to fund the entire sector programme rather than supporting separate projects. They are generally linked to macro-economic policy  instruments, such as a Medium Term Expenditure Framework |

Notes: HeRWAI: Health Rights of Women Assessment Instrument; OECD: Organisation for Economic Co-operation and Development; UNGASS: UN General Assembly Special Session on Drugs**;** IPV: Intimate Partner Violence; SRH: sexual and reproductive health; PNG: Papua Nueva Guinea; CEDAW: Committee on the Elimination of Discrimination against Women; SWA: Sector Wide Approaches
