# Supplementary material for: Have Policies Tackled Gender Inequalities in Health? A Scoping Review
Source: Int J Environ Res Public Health. 2021 Jan 5;18(1):327. doi: 10.3390/ijerph18010327 (PMC7796005; doi:10.3390/ijerph18010327)
Supplement: Supplementary file 1 [file ijerph-18-00327-s001.zip › Supplementary files 1,2 and 3/Supplementary File 3. Recommendations.docx]

| **1st author, year** | **Aim** | **Methods** | **Time frame;**  **location** | **Policy area** | **Health problem** |
| --- | --- | --- | --- | --- | --- |
| **Forbes,**  **2011** [27] | To recommend strategies for developing, integrating, and modifying existing interventions and programs for the prevention of sexually transmitted diseases (STDs) and HIV/AIDS to make them more responsive to the needs of women and girls | Qualitative. Forum of professionals (health care providers, U.S. policy makers, and governmental and nongovernmental organizations). Participants at the Forum were charged with developing the specific recommendations/ strategies | 2010; U.S.A | Policies related to STDs and HIV/AIDS. (Health and No health policies) | Sexually transmitted diseases and HIV/AIDS |
| **Gupta,**  **2011** [31] | This paper reviews both knowledge and action on the impact of gender inequality on women in the context of HIV prevention and then lays out steps that can be undertaken by national governments to respond to gender inequalities and their impact on women’s risk of acquiring HIV | Qualitative analysis: case studies. | Not specified. Approx. 2002-2007; Sub-Saharan Africa, South Africa, Latin America and south-east Asia | Policies aimed at increasing the economic status of women, addressing violence against women and changing harmful gender norms (No health policies) | HIV |
| **Jonsson,**  **2006** [35] | To present the major findings of the Board’s* (National board of Health and Welfare) follow-up inquiry, whose objective was to review and analyse gender equity trends in health care, and to discuss conclusions to be drawn on how to promote a more gender sensitive and equitable health care | Mixed methods. Compares the situation in health after the 1996 proposals on gender and health. Data from multiple sources: surveys, meetings, reports, health and quality registers, literature review, etc. | 1996-2004;  Sweden | Health care, health education and health research policies. (Health policies) | Health |
| **Olinyk,**  **2014** [40] | To explore the perspectives of those involved in developing and implementing the "Agenda for accelerated country level action on women, girls, gender equality and HIV", to understand its strengths and limitations | Qualitative. Case studies. In-depth one-on-one interviews with professionals involved in the development and implementation of the Agenda. | 2010-2011; Global | Gender and HIV policies (Health and No health policies) | HIV-related health |
| **Panisello, 2016** [41] | To identify and analyse progress and difficulties in the development of the gender perspective in health policies in Catalonia | Qualitative: document analysis (health plans, public health plan, legislative measures and gender-sensitive government actions) and interviews with professionals | 2006-2015;  Catalonia, Spain | Health policies | Health |
| **Rottach,**  **2017** [43] | To deconstruct the various dimensions of gender and identify how programmes address these domains to improve health | Systematic review. Sub-analysis based on a broader systematic review | Not specified Approx. until 2013-2014; Asia, Middle East, sub-Saharan Africa and Latin America | Policies/ programmes aimed mainly to addressing gender norms, power dynamics, legal status and rights and access to health resources (No health policies) | Family planning, or maternal or child health |
| **Siliquini, 2009** [45] | To synthesize the determinants of gender inequalities through a narrative review that: (a) describes gender related variables that can create different levels of health; (b) describes key points that may assist in policy development and its reorientation towards gender differences; (c) debates potential approaches in understanding gender issues. | Review | 1997-2007;  Global | Health care policies and other kind of policies such as education policies and research policies (Health and No health policies) | Health |
| **Sinha,**  **2009** [29] | To contextualize and outline strategies for gender-equitable HIV services in rural India | Review | 1980-2008;  Rural India | Health care policies (Health policies) | VIH |
| **Sridharan, 2016** [47] | (1) To explore what it would take for an action plan to impact outcomes related to mainstreaming; (2) to explore whether mainstreaming gender is demonstrated in the action planning process at WHO. | Qualitative: Document analysis (WHO action plans) | Time frame and location N/A | Health policies | Health |
| **Theobald, 2017** [49] | To reflect on five key lessons learnt from the gender mainstreaming process and their implications for donors, policy makers and practitioners working in the NTD field, and highlight relevant lessons for promoting gender equity in NTD programmes. | Qualitative: Review, focus meeting and experience of the authors. Theoretical analysis and case study | Not specified time; Global (Countries where NTD are relevant) | Policies of any kind related to NTD.  (Health and No health policies) | Neglected Tropical Diseases (NTD) |
| **Theobald, 2005** [50] | To explore the impetus for and process of gender mainstreaming in SWAPs in the Ministries of Health in Uganda, Ghana, Malawi and Mozambique, and to outline some achievements and challenges. | Qualitative. Workshop for Ministerial gender focal points and NGO representatives working on mainstreaming gender in SWAPs | Not specified. Approx. 2003;  Africa: Uganda, Ghana, Malawi and Mozambique | Health sector policies (Health policies) | Health |

Notes: STD: Sexually transmitted diseases; NTD: Neglected tropical diseases; SWAPs: Sector Wide Approaches
